# Cethromycin pharmacokinetics and pharmacodynamics for single dose cure of *Plasmodium berghei* liver stages

**DOI:** 10.1128/aac.00215-25

**Published:** 2025-06-17

**Authors:** Grace Kennedy, Rachel M. West, Kristin Poti, Bryce Bobb, Matthew M. Ippolito, Mark A. Marzinke, Nikola Kaludov, David J. Sullivan

**Affiliations:** 1 W. Harry Feinstone Department of Molecular Microbiology and Immunology, Johns Hopkins Bloomberg School of Public Health 25802 , Baltimore, Maryland, USA; 2 Clinical Pharmacology, Johns Hopkins University School of Medicine, Johns Hopkins University 1466, Baltimore, Maryland, USA; 3 AliQuantumRx Inc, Baltimore, Maryland, USA; The Children's Hospital of Philadelphia, Philadelphia, Pennsylvania, USA

**Keywords:** cethromycin, chemotherapy, antimalarial agents, liver stage, blood stage

## Abstract

Cethromycin combines a quinoline nucleus and a macrolide for broad-spectrum antibacterial and antiprotozoan activity. Here, we characterized the murine pharmacokinetics and *Plasmodium berghei* lifecycle stage pharmacodynamics for the cethromycin base. Liver pharmacokinetic studies in mice show peak mM drug concentrations in the liver with 20 hour sustained levels above 10 μM. Peak concentrations in the liver were double the lung and about 440 times that of plasma. Immunofluorescence imaging of *in vitro* cethromycin-treated infected hepatocytes shows complete ablation of the apicoplast. We observed complete cure of *P. berghei* liver-stage infection by a single oral dose of 60 mg/kg in mice, which is equivalent to the 5 mg/kg human dose of 300 mg a day used in bacterial pneumonia studies. Cethromycin at 60 mg/kg daily for 7 days was curative in the high parasitemic *P. berghei* mouse model. Both mosquito membrane feeding of *Plasmodium falciparum* gametocytes incubated with 20 μM cethromycin and oral dosing in mice demonstrated no decrease in oocyst numbers. Cethromycin has been evaluated for efficacy against bacterial pneumonia in more than 5,000 patients with good safety profiles. Cethromycin has potential for rapid clinical development for casual malaria prophylaxis and possibly radical cure of dormant liver *Plasmodium vivax*.

## INTRODUCTION

Cethromycin is a ketolide class macrolide (erythromycin base with a single sugar and quinoline unsubstituted nucleus replacing the cladinose saccharide) with broad-spectrum antibacterial, mycobacterial, and antiprotozoal activity developed for community-acquired pneumonia (1). The drug was safe, but not superior to clarithromycin in a large phase 3 outpatient study of more than 1,000 participants (2), for which the new drug application was later denied for the nonsuperior efficacy results by the Food and Drug Administration (FDA) (1, 3). Cethromycin binds to bacterial 23S ribosomal RNA of the 50S ribosome subunit, which stops polypeptide amino-acid addition, terminating protein synthesis and growth (4). Cethromycin specifically binds domains II and V in the wall of the polypeptide exit tunnel (1). While ketolides like cethromycin have greater activity than azithromycin against some bacterial species (5), both are unable to overcome high-level *erm* resistance (6–8).

The macrolide class has demonstrated delayed death activity *in vitro* and *in vivo* against protozoan apicomplexans like *Toxoplasma* (9) and *Plasmodium* (10–12). The plant-like apicoplast organelle is essential in the production of isoprenoids for blood-stage parasites. Previous work has shown that isoprenoid supplementation with azithromycin treatment results in loss of the extrachromosomal apicoplast genome and of the organelle in erythrocyte stages (13).

During the liver stages of malaria infection, the macrolide molecule has also been shown to delete the apicoplast and the extrachromosomal apicoplast genome (14). Thus, while the liver stages of malaria infection progress, loss of the apicoplast prevents infection of erythrocytes and transition to the blood stage (15). Azithromycin and doxycycline can cure mouse liver stages (16), but in controlled human malaria challenge studies, only 4 of 10 patients treated with azithromycin (17) and 8 of 12 patients treated with doxycycline (18) were cured after 7 days of liver-stage treatment initiated on sporozoite challenge.

In a previous report, we described liver-stage *Plasmodium* inhibition in the quantitative PCR mouse liver-stage sporozoites challenge model, with single low oral doses of cethromycin at 15, 25, and 50 mg/kg, because of access to only a few milligrams of newly synthesized cethromycin (19). Here, we report more extensive dose ranging and schedules for cethromycin pharmacokinetics (PK) and pharmacodynamics in murine malaria liver, blood, and gametocyte stage models.

## RESULTS

### Pharmacokinetics

**Fig 1:**
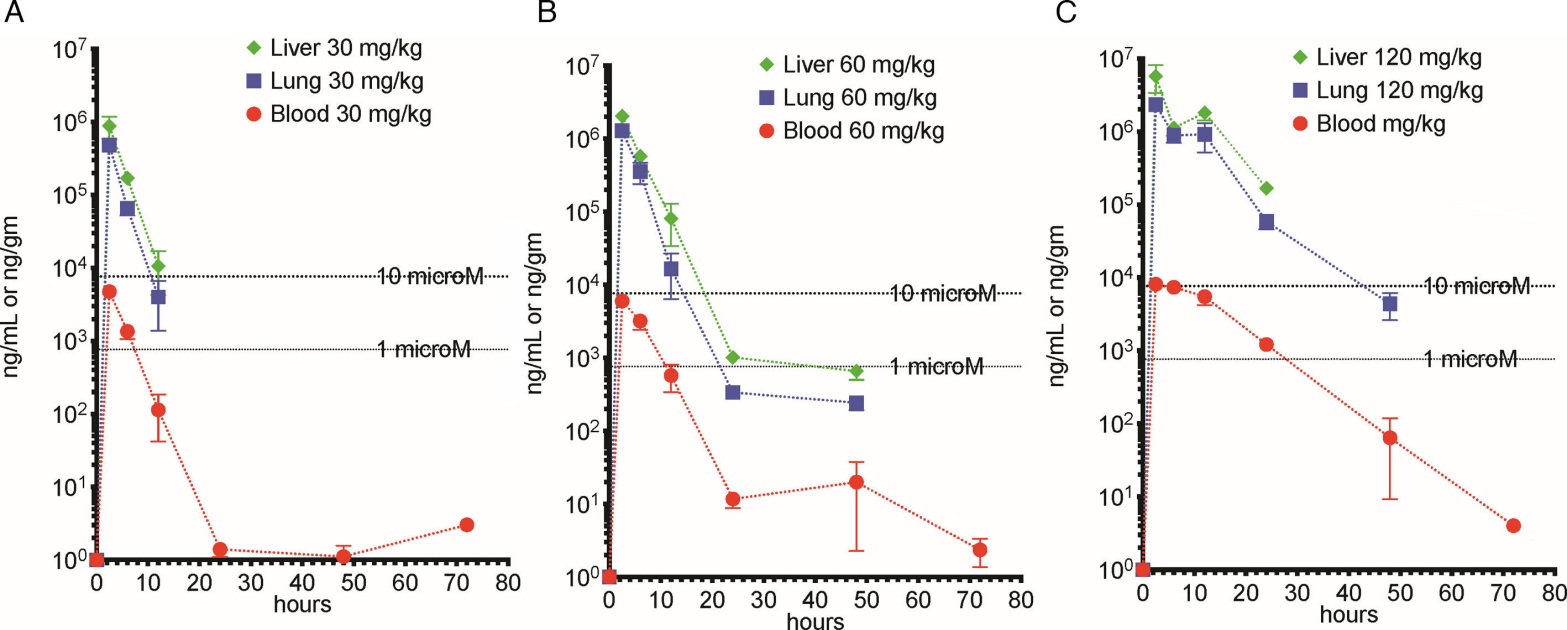
Liver, lung, and blood pharmacokinetics. Single oral cethromycin doses were given to groups of three mice with anticoagulated blood, liver, and lung harvested, and frozen on dry ice after separation of plasma. (**A**) 30 mg/kg, (B) 60 mg/kg, and (C) 120 mg/kg. Graphs show the mean and standard deviation. Dotted lines are 1 and 10 μM at 765 and 7,650 ng/mL, respectively.

We compared cethromycin concentrations in liver, lung, and plasma with single oral doses in mice at 30, 60, or 120 mg/kg, which is allometrically equivalent to 150, 300, and 600 mg total oral dose in humans (20). The maximum concentration (*C*_max_) in the liver was double that of the lung and approximately 440 times higher than plasma concentrations. The 765 g/mole cethromycin molecular weight indicates 1 μM levels at 0.76 μg/mL or 0.76 μg/g in the liver. The time above 1 μM in the liver was 24 hours for the 60 mg/kg dose. Plasma concentrations never exceeded 10 μM and stayed above 1 μM for 12 hours for the 60 mg/kg dose, which is allometrically equivalent to a single 300 mg oral dose in humans (Fig. 1). The tissue disposition of cethromycin measured as *C*_max_, area under the curve (AUC_0-72_), and elimination half-life (*t*_1/2α_) are shown in Table 1.

**TABLE 1:** Tissue distribution of cethromycin after single oral doses in mice (*n* = 3 per group)^*a*^

Tissue	Dose (mg/kg)	*C*_max_ (ng/mL)	AUC_0-72_ (ng hours/mL)	*t*_1/2α_ (hours)
Plasma	30	4,767 ± 754	18,682 ± 2,216	1.77 (1.56–2.03)
Plasma	60	5,994 ± 700	34,429 ± 3,572	2.32 (2.40–2.69)
Plasma	120	8,297 ± 604	117,134 ± 9,894	7.53 (5.82–10.66)
Lung	30	481,883 ± 45,681	1,502,310 ± 122,398	1.90 (1.58–2.39)
Lung	60	1,291,870 ± 189,700	4,856,050 ± 616,430	1.70 (1.48–2.00)
Lung	120	2,322,030 ± 83,339	17,565,300 ± 2,061,010	4.21 (3.31–5.80)
Liver	30	1,098,800 ± 253,711	3,585,590 ± 596,799	1.85 (1.61–2.17)
Liver	60	2,063,840 ± 74,153	8,573,030 ± 451,875	2.02 (1.85–2.23)
Liver	120	6,221,820 ± 1,501,440	37,067,800 ± 4,292,660	4.73 (3.64–6.74)

^*a*^ Mean and standard deviation of maximum concentration (*C*_max_) and area under the curve (AUC_0-72_) are shown. The 24 hour half-life (*t*_1/2α_) was estimated from linear regression of the log-transformed data with 95% confidence intervals shown in parentheses.

### Liver-stage apicoplast disruption

**Fig 2:**
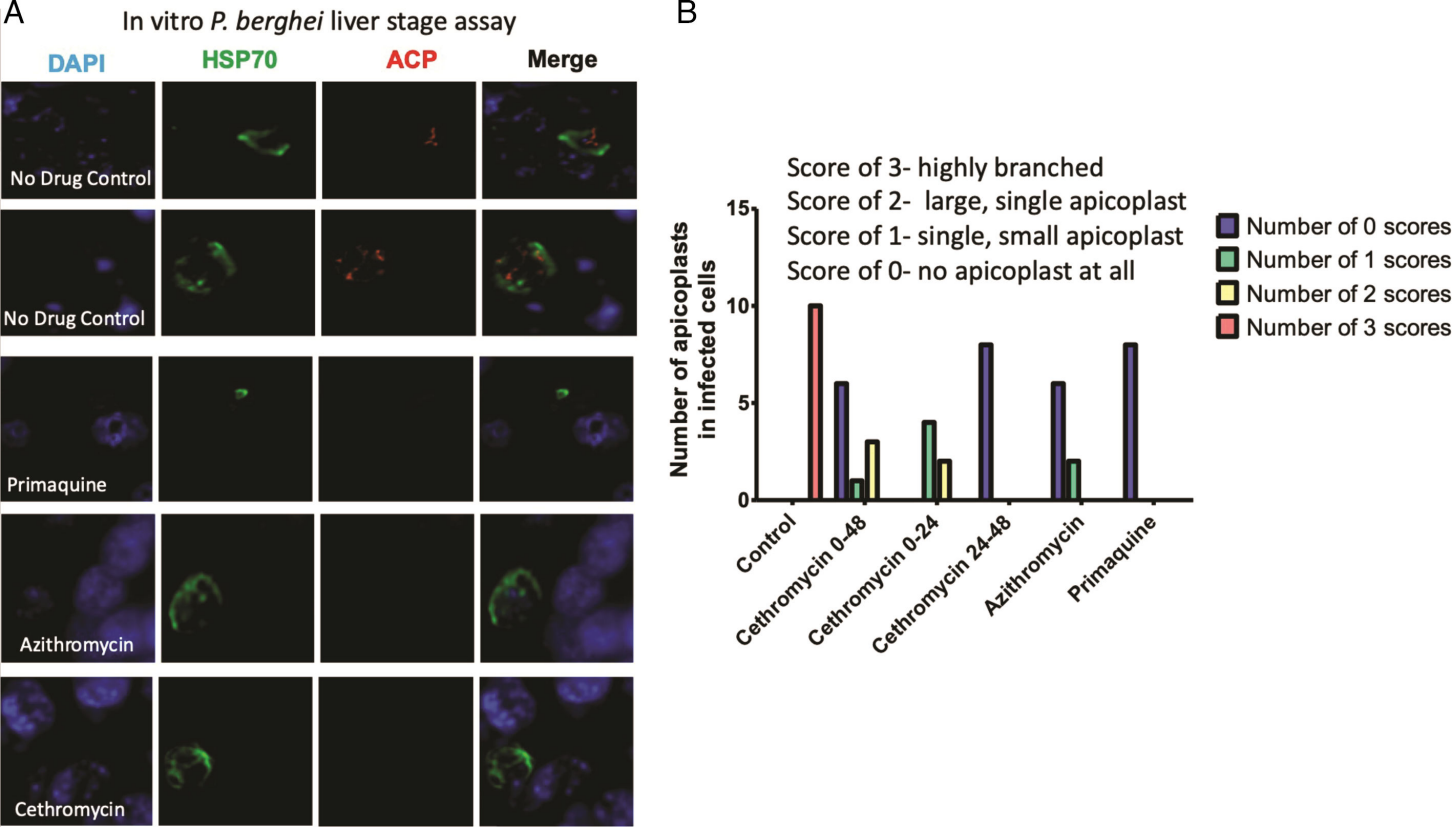
Hepatocyte *Plasmodium berghei* apicoplast imaging after drug treatment. Drugs at 20 μM were incubated 2 hours after sporozoite Hepa-1 hepatocyte invasion for 48 hours at which time cells were fixed for immunofluorescence. (**A**) Representative immunofluorescent images at 48 hours stained for nuclei with 4',6-diamidino-2-phenylindole (DAPI), cytosol with *P. berghei* Hsp70 and the apicoplast with acyl carrier protein (ACP). (**B**) Segregation of number size and branching of the apicoplast in hepatocytes under experimental conditions with the addition of cethromycin incubated for the first 24 hours and last 24 hours in addition to 48 hour drug incubations. Experiments were done in biological duplicate on two different dates.

We investigated maturation of the liver-stage *Plasmodium berghei in vitro* in 48 hour hepatocyte cultures treated with primaquine, azithromycin, and cethromycin exposure at 20 mM resulted in parasites with predominately no observed apicoplasts. Detailed counting of infected hepatocytes noted that those exposed to primaquine had parasites with no apicoplasts observed in hepatocytes, but single dysmorphic apicoplasts were present in parasites in cethromycin-treated and azithromycin-treated hepatocytes. In the controls, all apicoplasts were highly branched, indicating normal hepatocyte development of the parasite. The heat shock protein (hsp) 70 reporter also indicated almost normal parasite development with azithromycin and cethromycin exposure nearly indistinguishable from control activity, while primaquine-exposed parasites contained small pyknotic *P. berghei* Hsp70 signal (Fig. 2).

### Liver-stage pharmacodynamics

**Fig 3:**
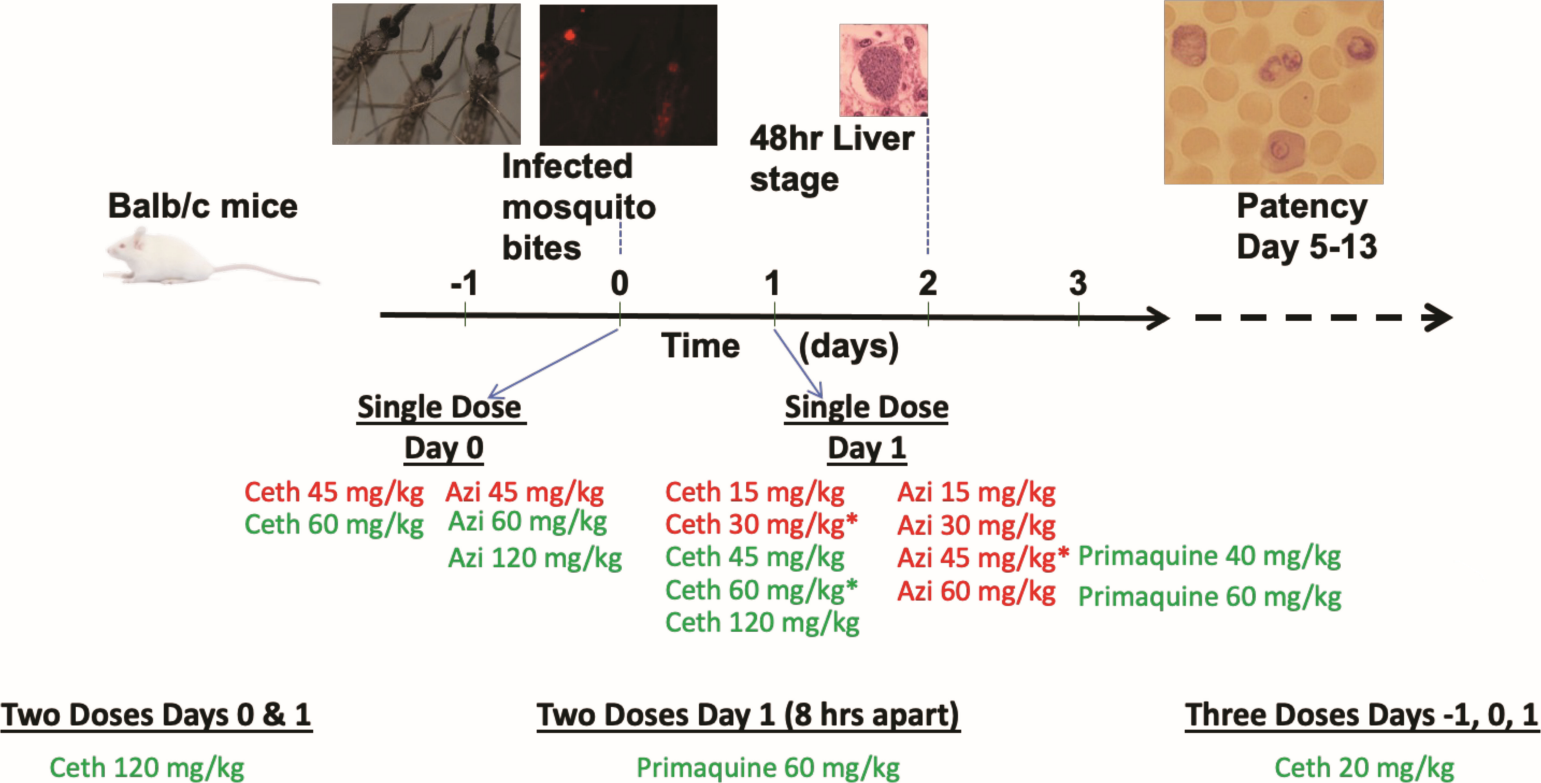
Schema for liver-stage dosing schedule with drugs dosed before, during, and after 48 hours *P*. *berghei* liver stage. Infected mosquitos were sorted with fluorescent microscopy for mCherry sporozoites, and 5–7 mosquitoes were fed on anesthetized mice. Patency was measured by Giemsa blood film for 16 days after sporozoite infection. Red labeled drugs indicate partial or all mice patent, and green labeled drugs indicate zero patent mice after liver-stage infection.

In the 3,000 sporozoite tail vein injection murine liver-stage model, repeated on three separate occasions in groups of three mice each, all nine controls had patent bloodstream parasites, while none of the nine mice treated with a single oral 60 mg/kg cethromycin dose 24 hours after sporozoite injection were patent. Utilizing a more natural lower dose of sporozoites with 5–8 infected mosquito bites, all controls were positive in the 10 separate biological replicates at different dates for dose ranging and timing experiments summarized in Table 2 and Fig. 3 with three mice in a group. The 60 mg/kg cethromycin dose was repeated as the relevant dose to the 300 mg total used in the human pneumonia studies. Partial curative doses were also more likely to be repeated in different biological experiments. Both azithromycin and cethromycin dosed 2 hours after mosquito infection were curative after a 60 mg/kg single oral dose, while 45 mg/kg was partially curative with a 2 day extension in days to patency. Azithromycin given 24 hours post-infection resulted in partial protection: 30 mg/kg (3/6 mice patent), 45 mg/kg (1/3 patent), and 60 mg/kg (3/6 patent), with a 3-day delay in patency observed at 30 mg/kg and 60 mg/kg. For cethromycin at 30 mg/kg (1/9 patent), 45 mg/kg (0/3 patent), and 60 mg/kg (1/9 patent), more mice were cured with patency delayed by 4 and 7 days over control patency of 6 days. Cethromycin 20 mg/kg in three separate doses on days −1, 0, and 1 was curative in all mice (*n* = 3). C57Bl/6 mice are more permissive/susceptible to murine malaria liver stages than BALB/c and have higher liver loads given the same inoculum. In C57Bl/6 mice, a single oral dose of 45 mg/kg azithromycin had 1 of 3 patent mice (delayed to 13 days), while at the same dose of cethromycin, 0 of 3 were patent.

**TABLE 2:** Liver-stage patency following mosquito bite sporozoite infection in 10 separate biological replicates at different dates for dose ranging and timing experiments with three mice in a group

Mouse	# Mosq.	Exp. #	Drug groups	Dose, mg/kg	Day of dose	Number pos/total	Average day to patency
BALB/c	7	8	Azithromycin	45	0	3/3	8
BALB/c	7	4, 6	Azithromycin	60	0	0/6	n/a^*a*^
BALB/c	7	4	Azithromycin	120	0	0/3	n/a
BALB/c	7	8	Cethromycin	45	0	1/3	8
BALB/c	7	4	Cethromycin	60	0	0/3	n/a
BALB/c	7	8	Azithromycin	15	1	2/3	7
BALB/c	7	8, 9	Azithromycin	30	1	3/6	9
BALB/c	7	9	Azithromycin	45	1	1/3	13
BALB/c	5	2, 6	Azithromycin	60	1	3/6	9
BALB/c	7	4, 5, 7, 8	Cethromycin	15	1	5/12	8
BALB/c	7	6, 8, 9	Cethromycin	30	1	1/9	10
BALB/c	7	9	Cethromycin	45	1	0/3	n/a
BALB/c	5	1, 2, 3	Cethromycin	60	1	1/9	13
BALB/c	5	1	Cethromycin	120	1	0/3	n/a
BALB/c	5	2	Primaquine	40	1	0/3	n/a
BALB/c	5	2	Primaquine	60	1	0/3	n/a
BALB/c	7	3	Cethromycin	20	−1, 0, 1	0/3	n/a
BALB/c	5	1	Cethromycin	120	0, 1	0/3	n/a
BALB/c	5	1	Primaquine	60	1, 1	0/3	n/a
BALB/c	7	1–9	Untreated	n/a		27/27	6
C57Bl6	7	9	Azithromycin	45	1	1/3	13
C57Bl6	7	9	Cethromycin	45	1	0/3	n/a
C57Bl6	8	9	Untreated	n/a		3/3	5

^*a*^ n/a-not applicable.

### Blood stage

**Fig 4:**
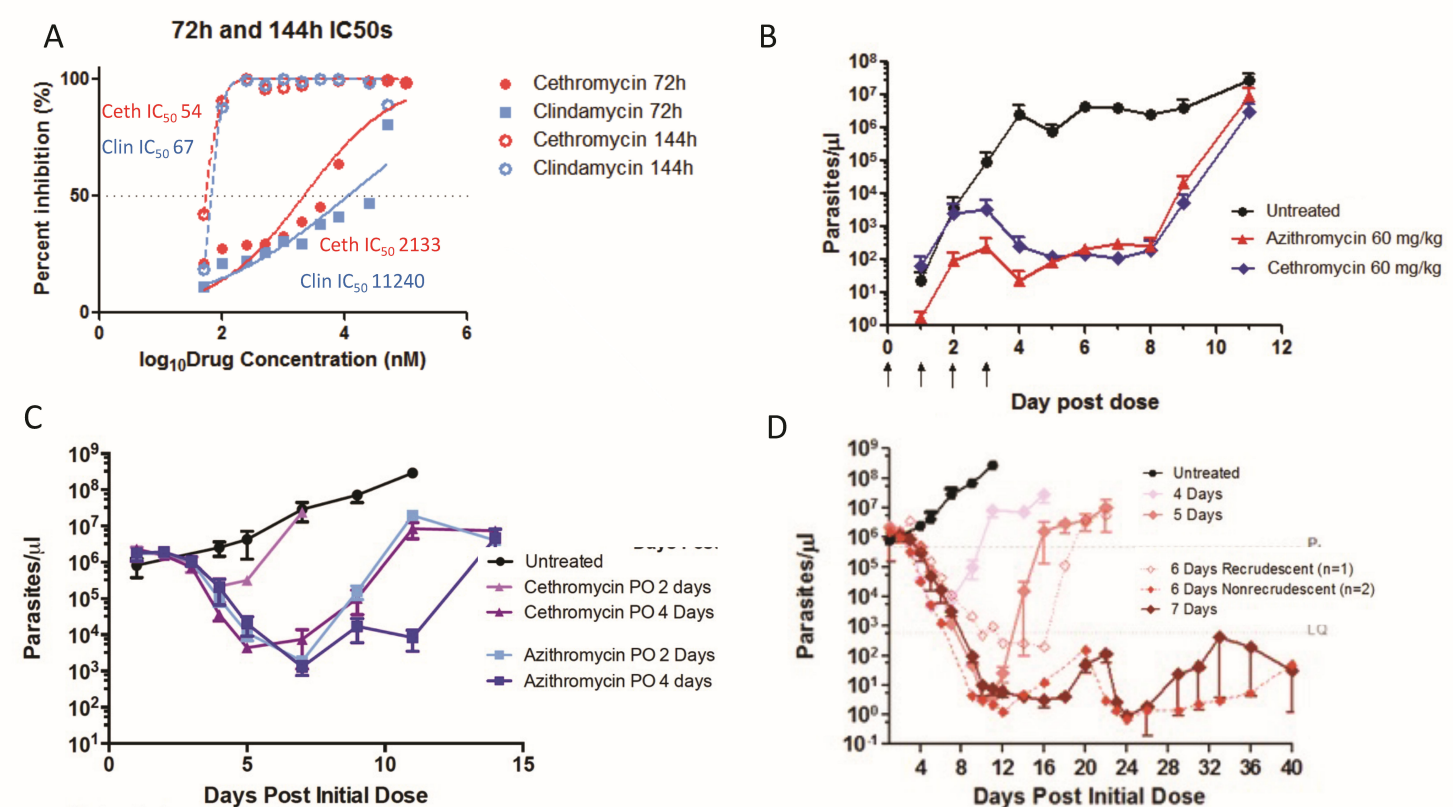
Blood-stage inhibition. (A) *P. falciparum* inhibition: continuous 72 hour and 144 hour *P*. *falciparum* 3D7 drug incubations with cethromycin and clindamycin. Assays performed in biological duplicates with technical triplicates. (**B**) *P. berghei* blood-stage 4 day suppression test with drugs starting 24 hours after inoculation for 4 days. (**C**) High parasitemia cytocidal test with 2 and 4 days of oral cethromycin and azithromycin. (D) High parasitemia test model with 4, 5, 6, and 7 days of cethromycin. One of three mice recrudesced with 6 days of dosing, while none recrudesced after 7 days of dosing. Female BALB/cj mice (*n* = 3) were inoculated with approximately 1 million infected erythrocytes from a donor mouse 4–5 days before drug dosing at about 1 million parasites per μL. Data are presented as mean ± standard error of the mean (SEM).

**Fig 5:**
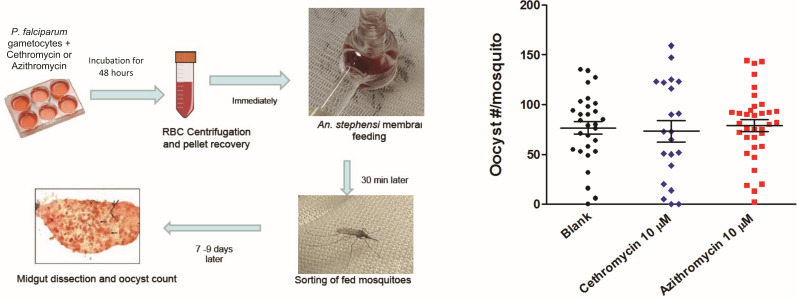
Late stage gametocyte to oocyst test. (A) Schema for late stage IV and V gametocytes on day 15 were dosed with 10 μM drug for 48 hours, cells were washed and immediately placed in membrane feeding apparatus, after which fed mosquitoes were sorted, and the number of oocysts per mosquito was counted. At least 20 mosquitoes were counted.

We measured the efficacy of cethromycin and clindamycin in *Plasmodium falciparum* isolates 3D7. The 72 hour IC_50_s were in the low μM range, while the 144 hour assays indicated 100-fold more potency to near 60 nM IC_50_ (Fig. 4A). In the murine *in vivo* 4 day suppression test, drug doses started after a low (1 million) parasite intraperitoneal inoculum. Both azithromycin and cethromycin at 60 mg/kg showed a similar >99% inhibition (more than 3 log difference in parasite number) on day 4 but both subsequently rebounded to one million parasites per μL on day 11 post-treatment (Fig. 4B). In the high-parasitemia model with 1 million parasites per μL, azithromycin showed greater parasite reduction than cethromycin at 2 and 4 days of oral drugs (Fig. 4C). In the same high parasitemia model, 6 days of cethromycin was partially curative (2/3 mice), while 7 days cured all mice (Fig. 4D). Investigation of cethromycin and azithromycin activity against late stage IV and V gametocytes, where 15-day-old gametocyte cultures were incubated for 48 hours with 10 μM drugs, followed by transmembrane feeding, noted similar oocyst numbers and infection rates (Fig. 5).

## DISCUSSION

In this work, we show that cethromycin demonstrates potent causal prophylactic activity against *P. berghei* malaria liver stages with superior cure rates compared to azithromycin at equivalent doses, while exhibiting typical macrolide delayed-death activity in blood stages and no significant effect on late-stage gametocytes. This activity profile aligns with the known mechanism of macrolide antimalarials, where the inability of the apicoplast to successfully replicate results in parasite death in the second generation of parasites and leads to the phenomenon of delayed generational death (21). Macrolides target both the *Toxoplasma* and *Plasmodium* apicoplast where the tissue stages appear to progress normally, but the progeny all are nonviable and lack functional apicoplasts for isoprenoid production (13). Cethromycin cures liver-stage malaria in mice with a single low dose of 60 mg/kg (human equivalent dose of 300 mg). Three doses of 20 mg/kg in mice over 3 days, which is equivalent to a 300 mg total dose in humans, were also curative. We compared the same dose of a related macrolide in widespread clinical use, azithromycin at 60 mg/kg, and this was not curative. The delay to patency was more prolonged in the breakthrough cethromycin mice compared to azithromycin-treated mice. Delay in patency usually correlates to lower liver loads in the *P. berghei* model (22). Primaquine was curative at a single dose of 40 mg/kg. The work of Milner et al. (23) looked at the day of dosing and dose for primaquine and tafenoquine in *P. berghei* liver stages, noting cure with 20 mg/kg on day 0 of sporozoite inoculation, whereas 40 mg/kg primaquine was noncurative dosed 24 hour after sporozoite infection for all five mice. Tafenoquine at 20 mg/kg was curative on either day 0 or day 1 as a single dose. Primaquine at 20 mg/kg is equivalent to a 100 mg total human dose, which is a higher allometric dose in mice than in humans on a mg/kg basis. Human primaquine dosing for relapse is 0.5 mg/kg a day or 30 mg base total a day in humans for liver-stage activity.

We observed dose proportionality at the 30 and 60 mg/kg doses for plasma, lung, and liver drug concentrations, but the 120 mg/kg had a higher AUC or longer lag, which we hypothesize may be due to saturation of tissue compartments. Conte et al. noted in humans that, for the 150 mg versus 300 mg dose, the AUC_0-24_ went from 0.9 to 3 μg hours/mL (24). In neutropenic mice dosed with 25, 50, 100, and 200 mg/kg cethromycin, plasma cethromycin pharmacokinetics also suggest slower clearance after high doses, but an AUC (24) was not generated. Cethromycin in mice at 60 mg/kg, which is allometrically equivalent to the 300 mg single daily dose in humans, achieved maximum liver concentrations of nearly 3 mM and remained above 1 μM for 24 hours (20). Azithromycin murine liver concentrations dosed at 200 mg/kg were 36 μg/mL (48 mM) a day after dosing (25) and were 200 times that of serum. Another mouse publication noted a peak liver azithromycin concentration of 73 μg/mL (97 mM) after a 50 mg/kg dose with four times higher concentrations in the liver than in the lung (26). Here, we noted 2,063 μg/mL as the observed peak mouse liver concentration after the 60 mg/kg oral dose, which was measured 2.5 hours post-dosing. These data support other findings which show the greatest cethromycin deposition in the liver. Macrolides are ideally suited by pharmacokinetics to work on liver infections. In humans dosed with cethromycin 300 mg, the *C*_max_ was 0.5 μg/mL in serum and 55 μg/mL (73 mM) in the lung with *t*_1/2_ of 5 and 12 hours, respectively, translating to potential near mM liver levels in humans (1).

In separate experiments initiated by blood-stage infection and bypassing the liver, 7 daily oral doses were curative of a high blood-stage parasite density. This is comparable to the 7 days necessary to cure with artesunate—a first-line agent for severe malaria—in the murine mouse model (27). However, the slow parasiticidal activity of macrolides, which kill by delayed generational death, precludes their use as monotherapy for severe malaria, where rapid parasite reduction is necessary (28).

The demonstrated cure and an excellent therapeutic product profile of the 3-keto macrolide quinoline cethromycin suggest further investigation into the niche area of dormant liver-stage malaria. Recent work noted disruption of the apicoplast in both dormant *Plasmodium vivax* and the primate equivalent species *P. cynomolgi* (14) with the speculation that azithromycin might work in relapsing malaria. In this prolonged *in vitro* hepatic culture study, uninucleate, presumably dormant liver-stage *P. cynomolgi* exposed to 12 days of 10 μM azithromycin disrupted all apicoplasts (14). Note that cethromycin may reach mM liver drug concentrations in humans. However, azithromycin in a triangular human clinical test design of five patients was not active in the prevention of dormant *P. vivax,* with three relapses stopping the trial (29). In the *P. cynomolgi* model, clindamycin by itself or with subcurative primaquine did not stop relapse, but azithromycin with subcurative primaquine prevented relapse in one of two monkeys, but could not be distinguished from radical cure with primaquine alone in this model (30).

We hypothesize that the quinoline ring might give more liver-stage killing than azithromycin. The two current anti-hypnozoite 8-aminoquinolines, primaquine (31) and tafenoquine (32, 33), require glucose-6-phosphate dehydrogenase deficiency testing, which is an impediment (time, extra clinical visit, cost, and accuracy of point of care test) to widespread drug implementation (34–37). The target indication for cethromycin is the prevention of dormant *P. vivax* malaria after curative chloroquine or artemisinin combination treatment for primary *P. vivax* parasitemia. The INSPECTOR trial noted antagonism of tafenoquine with dihydroartemisinin-piperaquine for dormant *P. vivax* (38), which might be avoided with cethromycin’s different mechanism of action. Thus, cethromycin might be superior to daily doxycycline prophylaxis with shorter duration after exiting malaria-endemic countries . Advantages to cethromycin are single daily doses, high liver tissue concentrations, and broad antimicrobial activity. Next steps include validation of efficacy for dormant malaria in the *P. cynomolgi* model and good manufacturing practice and investigational new drug enabling preclinical studies for human malaria testing.

## MATERIALS AND METHODS

### Animals and parasites

Inbred, female 6–7-week-old wild-type BALB/c and C57Bl/6 mice were purchased from The Jackson Laboratory (Bar Harbor, ME, USA) and kept in the animal facility at the Johns Hopkins Bloomberg School of Public Health. All animal experiments were approved by the Johns Hopkins Animal Care and Use Committee, under protocol MO15H319. Sporozoite-infected mCherry *P. berghei* mosquitoes were used for liver-stage infections (39). *P. berghei* MRA-868 ANKA (*Pb*-ANKA-Luc) transgenic strain was used for all murine blood-stage infections. The *Pb*-ANKA-Luc parasite strain expresses a Green Fluorescence Protein (GFP)-luciferase fusion gene . NF54 *P. falciparum* parasites at 4% hematocrit were cultured as described (40).

### Drug preparation and dosing

Primaquine, quinoline, and erythromycin were from Sigma. Azithromycin (Astatech cat # N460720) and cethromycin (BDG Synthesis, lot 16822, Wellington, New Zealand) were solubilized in either Dimethylsulfoxide (DMSO) for *in vitro* assays or Phosphate-Buffered Saline (PBS)/10% EtOH/20 mM HCl (875 μL PBS + 100 μL ethanol + 50 μL 1 N HCl per mL) for oral dosing in mice. For all the experimental studies done, each drug solution was dosed at a volume of 200 µL per mouse.

### Drug measurements

Cethromycin concentrations in plasma and tissue were determined via liquid chromatography-tandem mass spectrometry by the Clinical Pharmacology Analytical Laboratory at Johns Hopkins. Briefly, cethromycin was isolated from plasma and methanol-homogenized tissue lysate via protein precipitation using a 0.45 µm Captiva filter plate (Agilent Technologies, Wilmington, DE, USA). Cethromycin (C_42_H_59_N_3_O_10_) peak area ratios were normalized to the structural analog telithromycin (C_43_H_65_N_5_O_11_), which served as an internal standard. Cethromycin and its internal standard were quantified on an API 4000 (plasma) or API 4500 (tissue homogenates) (SCIEX, Foster City, CA, USA) operated in positive ionization and selective reaction monitoring modes. Ion transitions monitored were *m/z* 766.5→158.2 for cethromycin and *m/z* 813.6→656.2 for telithromycin. The primary analytical measuring ranges for plasma and tissue assays were 0.1–2,500 ng/mL and 10–2,500 ng/sample, respectively. Tissue results were normalized to the weight of tissue analyzed. Assays were validated in accordance with regulatory recommendations.

### Liver-stage *in vitro* assay

Hepa-1–6 (ATCC CRL-1830) were grown to confluence in Dulbecco's Modified Eagle Medium (DMEM) with fungizone/10% penicillin/streptomycin and plated onto Permanox LAB-TEK slides. Approximately 50,000 *P*. *berghei* sporozoites were added per well. After 2 hours, the medium was washed with fresh medium containing drugs at 20 µM. At 24 hours after sporozoite infection, cells were washed with fresh medium alone or fresh medium with drugs. At 48 hours, the medium was removed, and cells were fixed with 4% paraformaldehyde for 15 minutes, washed three times with PBS for GFP-luciferase assays or in cold methanol for 15 minutes, dried, and stored at −20°C overnight for immunofluorescence. Antibodies used were mouse anti-*P*. *berghei* Hsp70 (2E6), provided by Fidel Zavala, and rabbit anti-Pb acyl carrier protein, provided by Dr. S. Prigge (Johns Hopkins University). Secondary antibodies were Fluorescein Isothiocyanate (FITC) goat anti-mouse and Alexa594 goat anti-rabbit. The liver-stage assay experiment was performed in biological duplicate on two different dates.

### Model of sporozoite infection

Female *Anopheles stephensi* mosquitoes were fed with mCherry *P. berghei*-infected blood for sporozoite development, as previously described (22, 39). Twenty days later, cages of 200 mosquitoes were sorted with a fluorescent microscope to segregate positive mosquitoes. Mice were infected with 5–7 mosquitoes, and patency was examined by Giemsa blood film microscopy starting from day 4 to day 16 post-infection.

### *Plasmodium falciparum* culture

Low passage 3D7 or NF54 *Plasmodium falciparum* parasites were cultured and synchronized as described (41). Cultures were incubated with RPMI-1640 with L-glutamine, 25 mM HEPES, 40 µM hypoxanthine, 25 mM sodium bicarbonate, and 10% O+ human serum (Interstate Blood Bank). All cultures, unless otherwise noted, were in 2% hematocrit with O+ red blood cells. Cultures were gassed with 5% O_2_/5% CO_2_/90% nitrogen and placed in 37°C incubators.

### *In vivo* cytocidal model of murine malaria

The rodent model used for all the experiments was BALB/cj female mice from the Jackson Laboratories aged at least 6 weeks old and weighed approximately 20 g each. For each study done, replicates of three mice were used for each drug dose regimen tested. *P. berghei* ANKA, 676m1cl1, green fluorescence protein-luciferase (PbANKA GFP-Luc) obtained from ATCC (catalog # MRA-868) constitutively expresses the luciferase at all stages in the life cycle. For each experiment, BALB/cj female mice were infected via intraperitoneal (i.p.) with approximately 100,000 erythrocytes infected with PbANKA GFP-Luc from a donor mouse between 5% and 10% parasitemia (in the first or second passage). For malaria blood-stage infections and drug responses, there are no sex differences in BALB/c mice. As male and female BALB/c mice display differences in tissue iron levels, female mice were used for consistency throughout all experiments (42).

### Blood-stage murine luciferase assay and analysis

During the drug treatment, 5 µL of blood was collected from the tail of each mouse at regular intervals and deposited into 45 µL of lysis buffer in a 96-well plate (29). Samples were stored at −80°C until processed. A total of 5 µL of blood/lysis buffer (whole blood equivalent of 0.5 μL) was transferred to a black, opaque 96-well plate, and 95 µL of luciferase buffer (20 mM tricine, 100 µM EDTA, 1.07 mM K_2_CO_3_, 2.67 mM MgSO_4_, 17 mM DTT, 250 µM ATP, 250 µM D-luciferin) was added. Luciferase activity was measured in the IVIS Spectrum *In Vivo* Imaging System and analyzed using Living Image version 4.4 software. The raw luciferase activity is expressed as radiant flux in photons/second. Total radiant flux was compared to parasites per well using GraphPad Prism 5 software.

### Mosquito transmission

Gametocytes were cultured as previously described (43). Gametocytes (mature stages IV–V) were counted by blood film on days 15–16. Gametocyte cultures were then incubated with fresh medium alone or fresh medium with 10 μM drug for 48 hours, with the medium replaced (with or without drug) after 24 hours of exposure. Cultures were washed three times with fresh medium, and then diluted with RBCs and O+ human serum to a gametocytemia of 0.05%–0.07%, 50% hematocrit, and kept warm at 37°C. Cultures were fed to 4–6 hour starved *Anopheles stephensi,* using the standard membrane feeding assay (43). After feeding, blood-fed mosquitoes were sorted and kept. Mosquitoes were maintained for 8 days and fed with 10% sucrose solution. On day 8, mosquitoes were dissected, and midguts were stained with 1% mercurochrome to count oocysts.

### Pharmacokinetics

PK data were collected at multiple time points from mice dosed orally with cethromycin at 30, 60, or 120 mg/kg from plasma, lung, and liver. For cross-tissue comparisons, tissue concentrations in ng/mg were converted to ng/mL by multiplying by the approximate tissue density (lung ~1,000 mg/mL; liver ~1,050 mg/mL). Noncompartmental analyses were performed using the *PKNCA* (version 0.11.0) package in R (version 4.4.1). Mean tissue concentration-time profiles were generated for each dose group and tissue, and PKNCA was used to compute *C*_max_ and area under the concentration-time curve (AUC_0-72_) via the linear-up/log-down trapezoidal method. To estimate variability, bootstrapped Non-Compartmental Analysis (NCA) analyses (*n* = 1,000 resamples) were performed for each tissue-dose combination. To capture active elimination kinetics, and given that concentration-time profiles showed two phases—a rapid initial decline over 24 hours (alpha half-life, *t*_1/2α_) and a very slow terminal phase >100 hours, likely reflecting tissue redistribution—we calculated *t*_1/2α_ by fitting a linear regression of the log-transformed data for the first 24 hours.

### Statistics

Comparisons between liver-stage groups in this study are qualitative only to characterize trends, and no statistical comparisons of groups were performed. The pharmacokinetic data are mean and standard deviation of three mice at each dose time point. The murine blood-stage parasitemia and oocyst numbers were graphed with mean and SEM.

## SUPPLEMENTAL MATERIAL

Supplemental material aac.00215-25-s0001.docx Fig. S1 and S2; Tables S1 and S2.

ASM does not own the copyrights to Supplemental Material that may be linked to, or accessed through, an article. The authors have granted ASM a non-exclusive, world-wide license to publish the Supplemental Material files. Please contact the corresponding author directly for reuse.
